# UV-Light Effects on Cytochrome *C* Modulated by the Aggregation State of Phenothiazines

**DOI:** 10.1371/journal.pone.0076857

**Published:** 2013-10-09

**Authors:** Carolina G. dos Santos, André L. Silva, Flavio L. Souza, Alexandre J. C. Lanfredi, Paolo Di Mascio, Otaciro R. Nascimento, Tiago Rodrigues, Iseli L. Nantes

**Affiliations:** 1 NanoBioMAv, Centro de Ciências Naturais e Humanas, Universidade Federal do ABC, Santo André, SP, Brazil; 2 Universidade de Mogi das Cruzes UMC, Mogi das Cruzes, SP, Brazil; 3 Centro de Engenharia e Ciências Sociais Aplicadas, Universidade Federal do ABC, Santo André, SP, Brazil; 4 Departamento de Bioquímica, Instituto de Química IQ, Universidade de São Paulo USP, São Paulo, SP, Brazil; 5 Grupo de Biofísica Molecular “Sérgio Mascarenhas”, Instituto de Física de São Carlos IFSC Universidade de São Paulo USP-São Carlos, São Carlos, SP, Brazil; Consejo Superior de Investigaciones Cientificas, Spain

## Abstract

The present study shows the factors that modulate the photodamage promoted by phenothiazines. Cytochrome *c* was irradiated with UV light for 120 min, over a pH range from 4.0 to 8.0, in the absence and in the presence of different concentrations of thioridazine (TR) and fluphenazine (FP). In the absence of phenothiazines, the maximal rate of a Soret band blue shift (nm/min) from 409 to 406 nm was obtained at pH 4.0 (0.028 nm/min). The presence of phenothiazines at the concentration range 10-25 µmol/L amplified and accelerated a cytochrome *c* blue shift (409 to 405 nm, at a rate = 0.041 nm/min). Above 25 µmol/L, crescent concentrations of phenothiazines contributed to cytochrome *c* protection with (maximal at 2500 µmol/L). Scanning electronic microscopy revealed the formation of nanostructures. The pH also influenced the effect of low phenothiazine concentrations on cytochrome *c*. Thus, the predominance of phenothiazine-promoted cytochrome *c* damage or protection depends on a balance of the following factors: the yield of photo-generated drug cation radicals, which is favored by acidic pH; the stability of the cation radicals, which is favored by the drug aggregation; and the cytochrome *c* structure, modulated by the pH.

## Introduction

**Figure 1:**
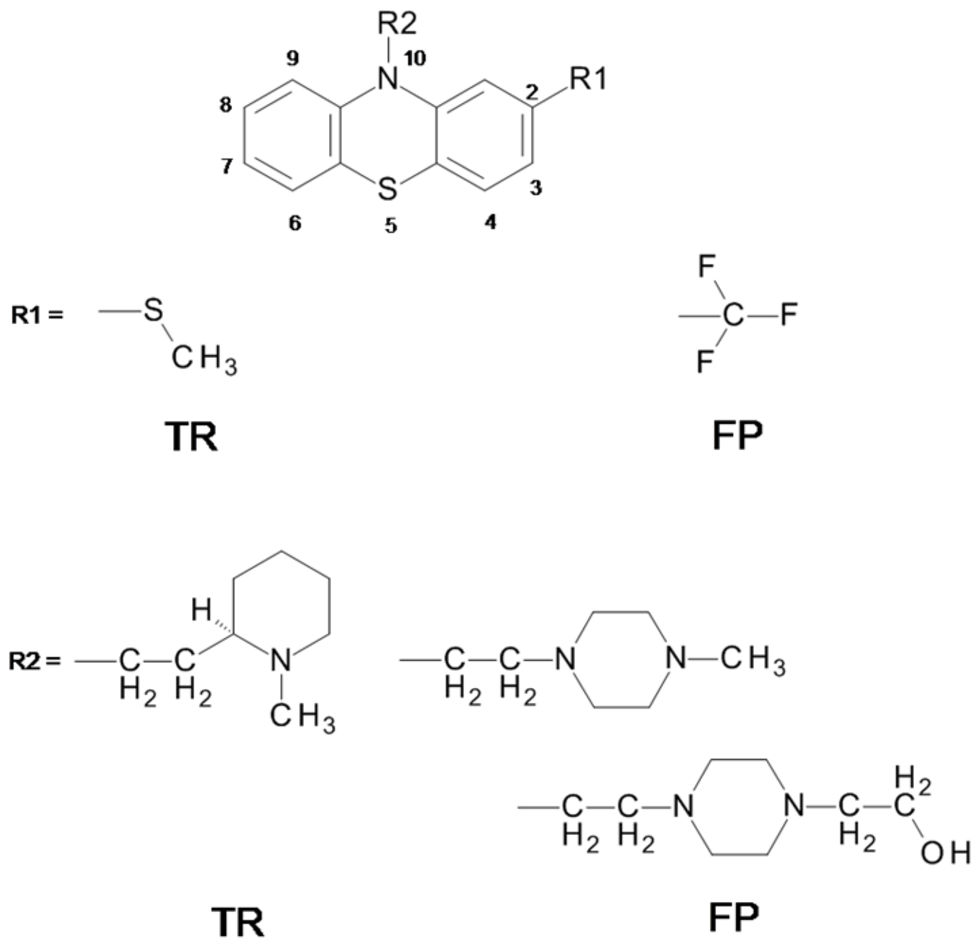
General structure of phenothiazines and substituents of the compounds used in the present study: TR, and FP.

Phenothiazines constitute a class of neuroleptic drugs, primarily used as antipsychotics since the early 1950’s decade. More recently, multiple other non-psychiatric effects have been described for drugs that can potentially be used for clinical applications [1-4]. The chemical structure of phenothiazines is a tricyclic ring constituted by two phenyl rings bound by sulfur and nitrogen atoms. The cyclic nucleus can be substituted at nitrogen 10 and carbon 2 (Figure 1). The phenothiazine substituents are correlated with the diversity of biological activities described for phenothiazines [5].

Phenothiazines exhibit certain interesting photosensitizing properties, which are responsible for the observed photosensitization in the skin of patients under therapy with these drugs [6-10]. During a photo-excitation process, the drug generates excited-states species and free radicals. In a biological system, these reactive species can cause damage in biomolecules such as lipids, proteins, and DNA [6-10]. Previously, it was demonstrated that the photo-excitation of the aggregate state of three phenothiazine derivatives (thioridazine (TR), trifluoperazine (TFP), and fluphenazine (FP) generates the corresponding cation radicals, that remained stable during hours [11]. However, TR, TFP, and FP cation radicals are not stabilized when the drugs are associated to lipid bilayers and promote oxidative damage in membranes [12]. Therefore, the pro-oxidant activity of phenothiazines is dependent of the stability of the cation radical. The cation radical stability is modulated by the aggregation state of the drugs that is dependent of the structure of the compound. The generation of phenothiazine cation radical results from UV light absorption, and thus the stability of the cation radical can potentially be associated to a photo-protection in this condition [13-15]. In addition, the phenothiazine nucleus (PHT) exhibited antioxidant properties when associated to mitochondrial membranes and liposomes. Studies using the quenching of excited states of 1-palmitoyl-2[10-pyran-1-yl)]-decanoyl-sn-glycero-3-phophocholine (PPDPC), incorporated in phosphatidylcholine/phosphatidylethanolamine/cardiolipin liposomes, demonstrated that PHT associates to membranes by hydrophobic interaction with lipid bilayers [13]. When associated to bilayers, 5 µM PHT was able to protect lipids and cytochrome *c* against pro-oxidant agents. PHT was also able to prevent mitochondrial permeabilization, which is promoted by peroxide and iron [13]. The association of phenothiazines with lipid bilayers has been also related to the capacity of these compounds in modulating multidrug resistance [16]. Similarly to free radicals, excited species are well known to promote oxidative damages in biomolecules [17]. In this regard, cytochrome *c* is a target for oxidative damages promoted by free radicals and excited species [18-20], and the repercussions are of great interest since this protein is decisive for the cell fate toward life or death [21-24]. Furthermore, cytochrome *c* maintains the native structure in a broad pH range (3 to 9) [25,26]and is a good model for studies involving pH effects promoted by chemical modifiers [19,27]. Therefore, singlet oxygen (O_2_ (^1^Δ_g_)) that mediates the photodynamic action of some drugs can promote alterations in cytochrome *c* structure and reactivity and influence the activation of caspases in the apoptosome [20]. On the other hand, the photodynamic generation of O_2_ (^1^Δ_g_) is also accompanied by free radical production [17,25,26,28–30]. The effect of photo-generated singlet oxygen (O_2_(^1^ Δ_g_)) (type II mechanism) and free radicals (type I mechanism) on cytochrome *c* structure and reactivity has been determined [19,28]. It has been demonstrated that above a pH range in which cytochrome *c* is in the native form, photo-generated free radicals induced a Soret band blue shift (from 409 to 405 nm). This is promoted by the conversion of the cytochrome *c*heme iron from its native low spin statewith rhombic symmetry (spin 1/2, g axially=3.07 and g radially=2.23), in which His18 and Met80 are the axial ligands to a high spin state with axial symmetry (spin 5/2, g axially=6.0 and g radially=2.0) formed by disruption of Met80 coordination [25,31-33]. Soret band bleaching, due to direct attack on the heme group, was only detected under conditions that favored free radical production, or in the presence of a less structured form of the protein (above pH 9.3). Cytochrome *c* with a Soret band at 405 nm (cyt*c*405) revealed oxidative modifications on methionine (Met65 and Met80) and tyrosine (Tyr74) residues. The damage of cyt*c*405 tyrosine residue impaired its reduction by diphenylacetaldehyde [27,34], but not by β-mercaptoethanol, which was able to reduce cyt*c*405 and generate cytochrome *c* Fe^2+^ in the high spin state (spin 2) [19]. Further studies revealed that cyt*c*405 is unable to trigger apoptosis [20]. Here, the studies concerning the photo-damage of cytochrome *c* were extended by investigating the effect of two phenothiazine drugs of clinical use: thioridazine (TR) and fluphenazine (FP) on the protein influenced by the pH and the aggregate state of the drugs.

## Materials and Methods

### Chemicals

Cytochrome *c* (horse heart, type III) and SDS were acquired from Sigma. The phenothiazine drugs like phenothiazine nucleus (PHT), thioridazine (TR), and fluphenazine (FP) were purchased from Aldrich. Diphenylacetaldehyde was purchased from Aldrich. Water was bi-distilled from an all-glass apparatus and further purified via a Millipore Milli-Q system. The universal buffer was prepared with sodium phosphate, acetic and boric acids according to reference [28,35]. The composition of universal buffer warrants efficient buffering at pH range 3 to 8 that was used in the present study.

### Generation of excited species and cation radicals by the photosensitization of phenothiazines

Samples were irradiated as function of the fixed excitation wavelength (λ = 254 nm) at 25°C in a quartz cuvette, in either deionized water or 5 mmol/L universal buffer. A compact 4W UVB lamp (UVGL-25 model, UVP Company, Upland, CA, USA)was used to irradiate the samples. The incident light intensity was set at 4 mWcm^-2^ determined using the equipment Laser Power Meter Model FieldMate with PowerMax thermal sensors at fixed distance (4 cm). Moreover, the cuvette was cooled by circulating water. Other additions are indicated in the figure captions.

### Purification of cytochrome *c* modified by reactive species

The samples containing cytochrome *c* with a Soret band peaking at 405 nm (cytc405) were purified with Chelex 100® and Centricon®, which exhibits a binding affinity to phenothiazines. Purification of phenothiazines with Chelex 100^®^ particles was monitored by spectrophotometry, indicating the disappearance of the phenothiazines’ absorbance spectrum.

### Electronic Absorption Spectroscopy

The electronic absorption measurements of cytochrome *c* were conducted in photodiode spectrophotometer (Shimadzu Scientific Instruments Inc., Columbia, MD) using quartz cuvettes of a 1 cm light path and a slit of 0.5 nm.

### EPR Spectroscopy

The direct EPR measurements of cytochrome *c* (100 µmol/L) were taken using an EPR Bruker system, ELEXSYS model E-580. The measuring conditions were as follows: gain, 5 x 10^3^; modulation amplitude, 1.0 mT (for heme iron); microwave power, 4 mW; temperature, 11 K; time constant, 1.28 ms; and conversion time, 81.92 ms.

The samples were introduced into an EPR quartz tube and precooled in liquid nitrogen before being placed in the microwave cavity at low temperature.

### CD and MCD measurements

The CD and MCD measurements were carried in a Jasco J-720 spectropolarimeter (Easton, MD, USA) using quartz cuvettes of a 0.1 cm optical path. For the MCD measurements, the magnetic field was 860 mT. Cytochrome *c* Fe^2+^ samples were prepared and kept in a nitrogen atmosphere during handling and analysis.

### Matrix-assisted laser desorption ionization (MALDI) time-of-flight (ToF) mass spectrometry

The MALDI-ToF MS analyses were performed using an Ettan MALDI-ToF Pro system equipped with a quadratic-field reflectron and a timed ion gate. Protein identification was conducted in reflectron mode, with positive ionization at 20 kV. The sample was, in this case, mixed with an equal volume of 50% acenitrile and 0.5% trifluoracetic acid, which was saturated with α-cyano-4-hydroxycinnamic acid. A 0.5 µL of the mixture (containing 8 pmol of protein) was loaded onto the stainless steel MALDI slides for analysis. The external calibration was performed before protein identification with AngIII and hACTH 18-36. The data were analyzed using the Ettan MALDI-ToF Pro software system.

### Scanning Electron Microscopy (SEM)

Samples containing cytochrome *c* and 2500 µmol/Lphenothiazines were irradiated and purified as described above. In the following, the samples were centrifuged, the supernatant discarded, the pellet transferred to a silicon plate and dried at 60°C for one hour. The morphologmorphology of the samples containing phenothiazine particles were obtained in a JEOL JSM-6701 F field emission scanning electron microscope. Size measurements were done using specific public domain software (ImageJ, Wayne Rashand, rsb.info.nih.gov/ij/). Particle size measurements were undertaken in 2 sample areas of 933 X 1,066 nm and 8000 X 6,400 nm according to the highest longitudinal dimension and were expressed using descriptive statistics (Microcal Origin 8.5, frequency counts).

## Results and Discussion

### Effect of the phenothiazine concentration and pH on the photo-oxidative modifications of cytochrome *c* structure

**Figure 2:**
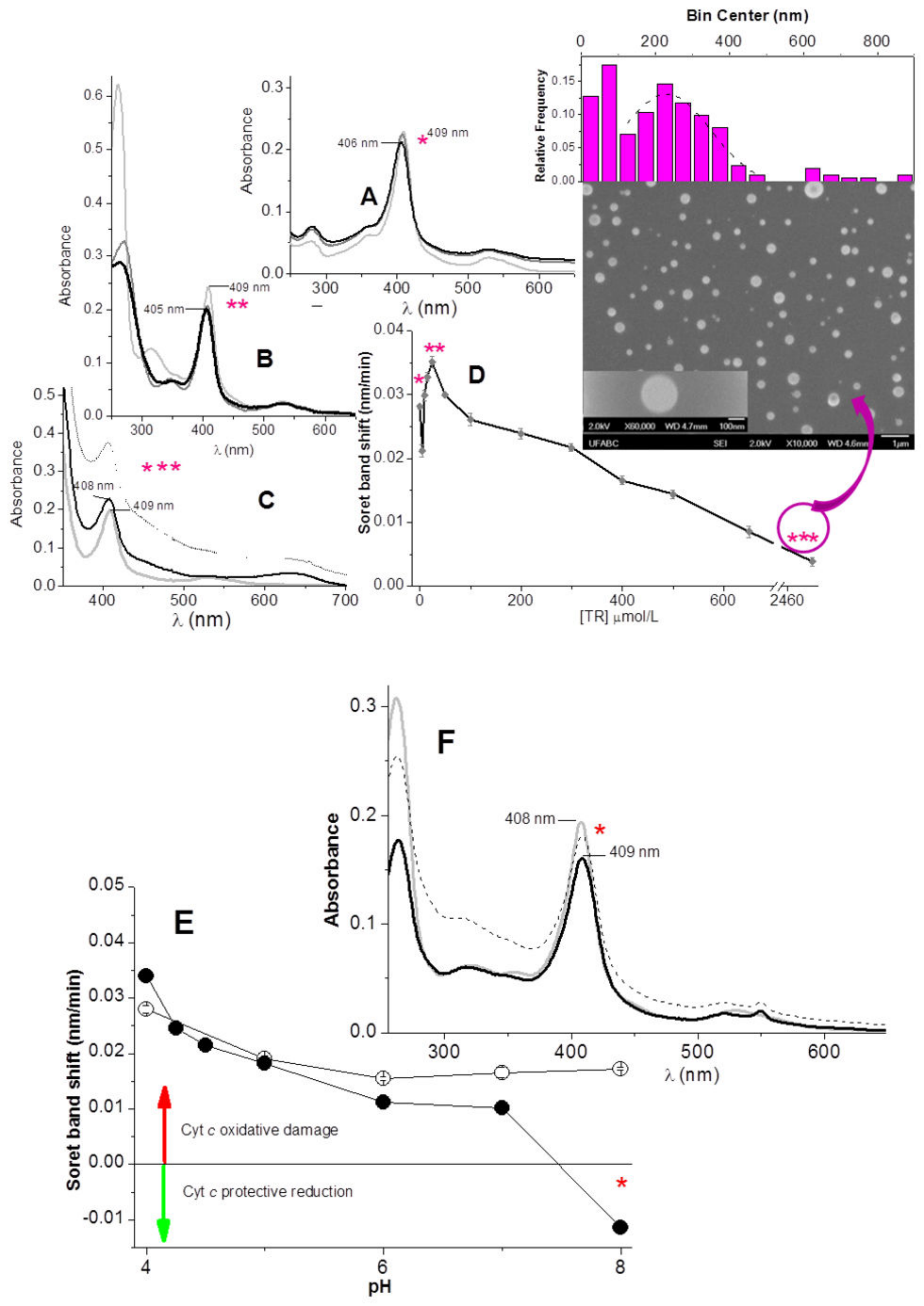
Oxidative modifications induced by a TR cation radical on cytochrome **c**.A - Spectral changes of cytochrome *c* before (light gray line) and after 30 (gray line), and 120 (black line) min UV light irradiation at 254 nm, at pH 4.0. **B** –The same conditions described for A in the presence of 25 µmol/L TR. **C** - The same conditions described for Ain the presence of 2.5 mmol/L TR. In this panel, the dashed line represents the original spectrum of cytochrome c before the subtraction of the turbidity sample contribution that resulted in the spectrum shown as a black line **D** – The effect of different TR concentrations on the rate of Soret band blue shift promoted by UV irradiation. In panel D, the number of asterisks indicates the corresponding spectra shown in panel A (*), B (**), and C (***), from which the data were extracted. The inset shows SEM images and the distribution of particle size obtained for the sample in the condition marked with three asterisks. **E** – The effect of pH on the rate of Soret band blue shift promoted by UV irradiation in the presence of 25 µmol/L TR. Filled black circles represent the results in the absence of TR and opened circles represent the presence of TR. In panel E, the asterisk in one data point indicates that the point was extracted from the corresponding spectrum shown in panel F. **F** – The same conditions described for B, at pH 8.0.In this panel, the dashed line represents the original spectra of cytochrome c before the subtraction of the turbidity sample contribution that resulted in the spectrum shown as a black line. Samples containing 3 µmol/L cytochrome *c* and TR, when indicated, were irradiated in a 5 mmol/L phosphate buffer at 25°C in the cuvette with a 4 mW/cm^-2^ as function of 254 nm of excitation at a distance of 4 cm from 1cm^2^ of a selected sample area.

Cytochrome *c* was irradiated at 254 nm with 4 mW cm^-2^ during 120 min, both in the presence and the absence of phenothiazines at pH = 4.0. In the absence of drugs, UV exposure led to a blue shift of the cytochrome *c*Soret band from 409 to 406 nm (Figure 2A). This spectral change had been previously characterized as associated to the loss of the heme iron sixth ligand, Met80, promoted by the oxidation by free radicals and excited species [19]. In the same conditions, cytochrome *c* was exposed to UV irradiation in the presence of the phenothiazines TR and FP. Figure 2B shows the alterations in the cytochrome *c* spectrum, promoted by UV irradiation in the presence of 25 µmol/L TR. The spectrum of cytochrome *c* in the presence of TR, obtained before irradiation (time zero), was typical of Fe^3+^, a low spin state native hemeprotein (light gray solid line). Upon irradiation, the spectral changes of cytochrome *c* at pH 4.0 were characterized by a blue shift and bleaching. The spectral changes of cytochrome *c*could be observed concomitantly with the spectral changes of TR previously assigned to the formation of sulfoxide and the derivative with the hydroxylated ring [11](Figure 2B). The aforementioned spectral changes of TR and cytochrome *c* were not detected when the sample was maintained in the dark conditions (not shown). Considering that TR and FP form aggregates that stabilize the respective cation radical derivatives [11], cytochrome *c* underwent UV irradiation in the presence of different concentrations of the phenothiazine. Figure 2C illustrates the differentiated effect of high concentrations of TR. In the presence of 2.5 mmol/L TR, cytochrome *c* was partially protected against the oxidative effect of UV irradiation. Figure 2 C also shows the TR 615 nm absorbance band in the spectrum of a mixture containing 5 µM cytochrome *c* and 2.5 mmol/LTR that was subjected to UV irradiation. TR cation radicals stabilized by the aggregation of the drug are related to the appearance of a band at 620 nm region while FP and the parent compound TFP cation radicals are associated to bands peaking at around 520 nm [11]. Figure 2D shows the rate of a Soret band blue shift plotted as a function of TR concentration. The blue shift of the cytochrome *c*Soret band, promoted by 120 minutes of irradiation at pH 4.0 and with a UV-lamp set at 4mW cm^-2^, occurred at rate = 0.028 nm/min. In the presence of 5 µM TR, a discrete decrease of the blue shift rate was observed. At the TR concentrations > 5 µmol/L≤ 25 µmol/L, cytochrome *c* damage was faster, and a larger Soret band blue shift was observed. The maximal damaging effect was observed in the presence of 25 µmol/L TR. In this condition, the Soret band peak shifted to 405 nm (Figure 2B) at a rate = 0.041 nm/min (Figure 2D). Above 25 µmol/L, increasing TR concentrations progressively protected cytochrome *c* against UV light-promoted oxidative damages. The maximal protective effect was obtained at 2.5 mmol/L. In the presence of 2.5 mmol/LTR, the Soret band blue shift was limited to a maximal extension of 2 nm (Figure 2C), which was attained at a rate = 0.005 nm/min. Thus at high concentrations, TR decreased eightfold the blue shift rate of the cytochrome *c*Soret band promoted by UV irradiation. In this condition, i.e., in the presence of 2.5 mmol/L TR, the increase of sample turbidity was also observed. The original spectrum obtained after a 120 min irradiation of cytochrome *c* in the presence of 2.5 mmol/LTR is shown as a dashed line in Figure 2C. The contribution of the turbidity (1/λ^3^) was subtracted for clarity, and the result was the spectrum shown as thick black line.

The results presented in Figure 2 A-D are consistent with the following events. The UV-filter effect of low TR concentrations is not enough to hamper the photo-damage of cytochrome *c*, and the photo-generation of TR cation radicals synergistically contribute to the photo-oxidative damages of the protein. At relatively higher TR concentrations, the formation of TR aggregates stabilizes the cation radicals generated by intense UV light absorption (more efficient UV-filter), and the net effect is cytochrome *c* partial UV-protection. The increase of sample turbidity observed in higher TR concentrations suggested the photo-induced formation of TR-derivative particles or aggregates. Therefore, samples containing cytochrome *c* in the presence of 2.5 mmol/LTR and submitted to a 120 min of UV light irradiation were imaged by SEM in order to elucidate events responsible by the turbidity increase. The SEM images of the abovementioned sample (inset of Figure 2D) show polydispersed spherical particles. Thesize measurements of the particles undertook in 2 sample areas of 933 X 1,066 nm and 8000 X 6,400 nm revealed a population of particles distributed in a Gaussian like profile. In this profile, it is predominant the particles with an average size of ~230 nm diameter (see histogram above SEM image). The concomitant presence of a significant population of size lower than 100 nm diameter raises the possibility that the larger particles resulted from the agglomeration of the primary smaller spherical particles. The mechanism of photo-induced formation of TR particles is currently under investigation in our laboratory. At this point, it is important to notice that the appearance of a broad absorbance band at the 600 nm region in TR-containing samples submitted to UV irradiation could result from the contribution of the phenothiazine oligomerization rather than the stable cation radical. Similar spectrum was previously observed by Abu-Abdoun & Ledwith, 1997, after photochemical polymerization of the phenothiazine nucleous (PHT) [28,36]. Therefore, the photo-induced oligomerization of TR from the reaction of cation radical derivatives might also prevent the contribution of TR radicals for cytochrome *c* damage induced by UV light irradiation.

**Figure 3:**
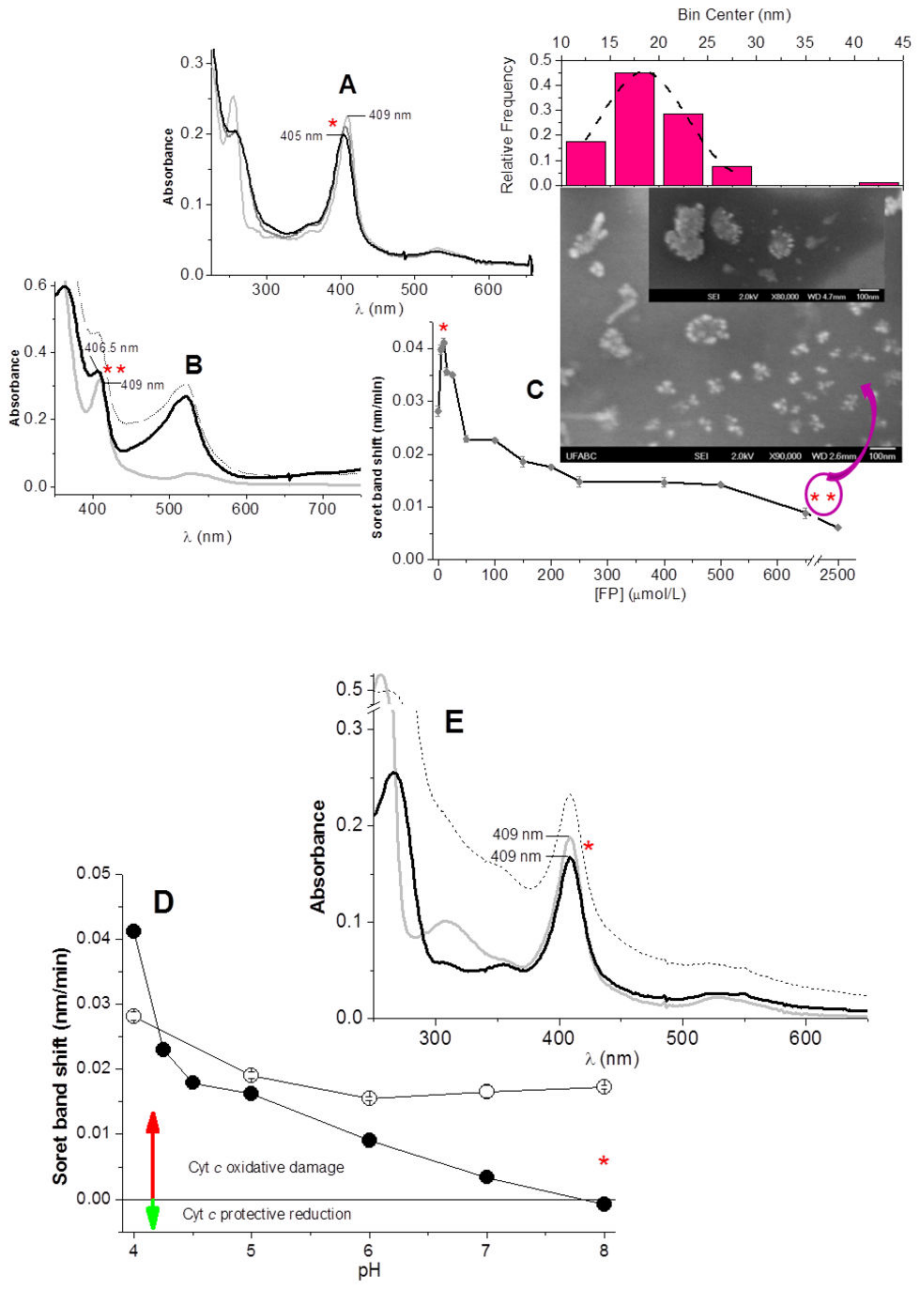
Oxidative modifications induced by FP cation radicals on cytochrome **c**.A - Spectral changes of cytochrome *c* before (light gray line) and after 30 (gray line), and 120 (black line) min UV light irradiation at 254 nm in the presence of 10µmol/LFP, at pH 4.0. **B** - The same conditions described for A in the presence of 2.5 mmol/LFP. In this panel, the dashed line represents the original spectra of cytochrome *c* before the subtraction of the turbidity sample contribution that resulted in the spectrum shown as a black line **C** – The effect of different FP concentrations on the rate of Soret band blue shift promoted by UV light irradiation. In panel C, the number of asterisks indicates the corresponding spectra shown in panel A (*) and B (**), from which the data were extracted. The inset shows SEM images and the distribution of particle size obtained for the sample in the condition marked with two asterisks. **D** – The effect of pH onthe rate of Soret band blue shift promoted by UV light irradiation promoted by 10 µmol/L FP. Filled black circles represent the results in the absence of FP and opened circles represent the presence of FP.In panel E, the asterisk in one data point indicates that was the point extracted from the spectra shown in panel E. **E** – The same conditions described for B, at pH 8.0. In this panel, the dashed line represents the original spectra of cytochrome *c* before subtraction of the turbidity sample contribution that resulted in the spectrum shown as a black line. Samples containing 3 µmol/Lcytochrome *c* and FP, when indicated, were irradiated in a 5 mmol/Lphosphate buffer at 25°C in the cuvette with a 4 mW/cm^-2^of UV light intensity as function of 254 nm of excitation at a distance of 4 cm from 1cm^2^ of selected sample area.

The effect of TR on UV-promoted damage on cytochrome *c* is modulated by the pH of the medium. The effect of pH was probed at 25 µmol/L TR (Figure 2E), which was the most efficient concentration to exacerbate the photo-damage of cytochrome *c* at pH = 4.0. The rate of the blue shift of the cytochrome *c*Soret band induced by UV-irradiation was determined over the pH range 4.0 to 8.0. In these conditions, native cytochrome *c* is in the low spin state and the drug (p*K*_a_ = 8.1) is predominantly (≥ 50%) protonated. Interestingly, the contribution for the damage was observed exclusively at pH 4.0, and the significant contribution for the UV protection was observed at above pH 5.0. Analysis of the cytochrome *c* spectrum during irradiation with the UV lamp in the presence of 25 µmol/L TR at pH 8.0 revealed that, in this condition, cytochrome *c* was partially reduced by the drug (Figure 2F), and the protein was totally protected against UV-induced oxidative damage. Similar results were obtained with FP (Figure 3). Figure 3A shows that the irradiation of cytochrome *c*, at pH 4.0 during 120 min with a 4 mW/cm^-2^ of UV light intensity (from 254 nm of excitation) in the presence of 25 µmol/L FP contributed to the UV-promoted cytochrome *c* blue shift. Conversely, the photo-induced damage was mitigated by the presence of 2.5 mmol/L FP (Figure 3B). In the presence of 25 µmol/L FP, the spectral changes indicative of the formation of FP derivative sulfoxide [11] were observed concomitantly with a Soret band blue shift. These results are consistent with the low stability of photo-generated free radicals of the drug. In the presence of 2.5 mmol/L FP, the partial protection of cytochrome *c* against UV light damage was coincident with the appearance of the 520 nm band assigned to cation radicals of the drug stabilized in the aggregates [11]. The plot of the rate of cytochrome *c* blue shift promoted by UV light irradiation as the function FP concentration revealed that relative low concentrations of this phenothiazine (5 and 10 µmol/L) also amplified and accelerated the photo-induced damage of cytochrome *c* (Figure 3C). In the presence of 10 µmol/L FP, the exposure of cytochrome *c* to 120 min UV irradiation at pH 4.0 promoted a Soret band blue shift to 405 nm at a rate of = 0.041 nm/min (Figure 3C). Above 25 µmol/L, increasing FP concentrations also protected cytochrome *c* against photo-induced damage. In these conditions, i.e. in the presence of FP aggregates, the derivative cation radical (band peaking at 520 nm) was also detected in a UV-visible spectrum (Figure 3B). In this condition, a significant increase of sample turbidity was detected after the irradiation of the sample and could also be attributed to the photo-induced formation of FP particles. Formation of FP nanoparticles was corroborated by SEM images and determination of size distribution (SEM image and particle size distribution as the inset of panel C, Figure 3). Differently of the results obtained for TR, the SEM image and the size distribution histogram determined for FP revealed a single population of FP nanoparticles with ~18 nm diameter as the predominant size. The high intensity band of FP cation radical of this sample (panel B of Figure 3) suggests a slower reactivity of this radical species toward the FP derived oligomer. In this condition, the lower yield of primary nanoparticles at the monitored time prevented the formation of secondary higher size particles. Similarly to TR, the effect of FP on UV-promoted damage on cytochrome *c* is also pH-dependent. Figure 3D shows the influence of pH on the capacity of 10 µmol/L FP to synergistically contribute to the photo-damage of cytochrome *c*. The rate of the blue shift of the cytochrome *c*Soret band induced by UV light irradiation was also determined over a pH range of 4.0 to 8.0. In the presence of FP, increased damage was observed only at pH 4.0, and significant protection was found above pH 5.0. Cytochrome *c* reduction was not significantly observed during UV light irradiation in the presence of 10 µ/L FP at pH 8.0 (2E), but protection against UV light -induced photo-damage was observed (Figure 3D).

### Characterization of cytc405 formed by photo-oxidative damage in the presence of TR

**Figure 4:**
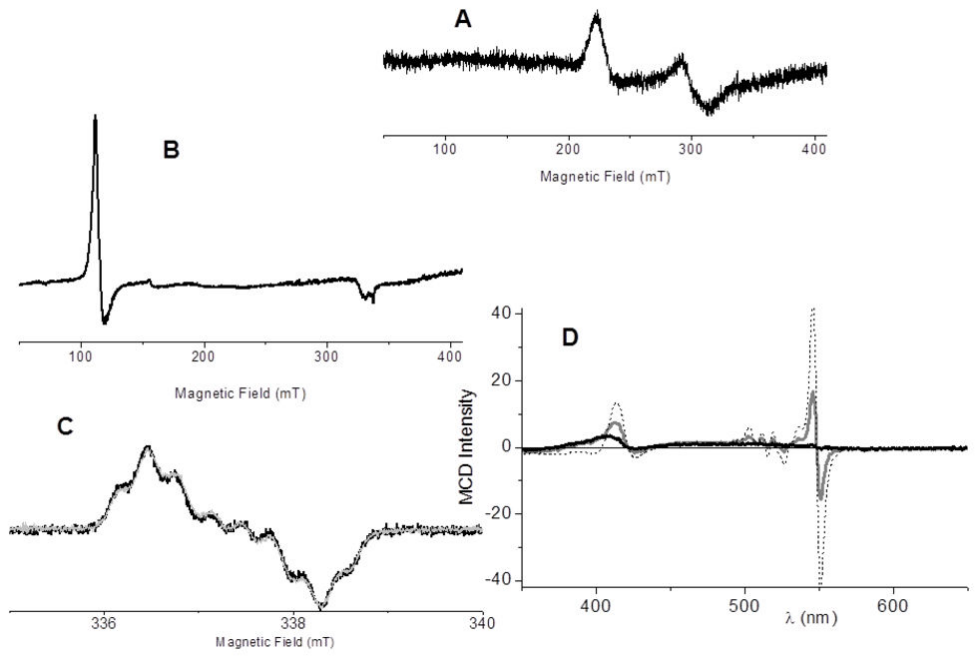
EPR and MCD analysis of cytochrome c modified by phenothiazinesA - EPR spectra of native cytochrome c; **B** - EPR spectra of cytochrome *c* 120 min irradiation in the presence of TR^+^. The spectra were obtained in the presence of 100 µmol/L cytochrome *c* and 200 µmol/L TR^+^ in a 5 mmol/L phosphate buffer, pH 7.4, at 11 Kelvin, 1 mT of field modulation, and 4 mW of microwave power in the X band. **C** – EPR spectra of TR at room temperature, which were obtained in the same conditions described for panel B. In this panel, the light gray line represents the spectrum obtained in the absence of cytochrome *c* and the black line the spectrum obtained in the presence of cytochrome *c*. Samples containing cytochrome *c* and TR were irradiated during 120 min in a 5 mmol L^-1^phosphate buffer, at 35°C in the cuvette with 4 mW/cm^-2^ of UV light intensity as function of 254 nm of excitation at a distance of 4 cm from 1cm^2^ of selected sample area. **D** - MCD spectra of cytochrome *c* obtained after 120 min of irradiation in the presence of TR (gray line). In this panel, the dashed line represents the spectrum of an identical concentration of cytochrome *c* (100 µmol/L), completely converted to the ferrous form by β-mercaptoethanol. The samples were irradiated, with 4 mW cm^-2^ of UV light intensity as function of 254 nm of excitation at a distance of 4 cm from 1cm^2^ of selected sample area, in 10 mmol/L universal buffer, pH 5.

The blue shift of the cytochrome *c*Soret band promoted by UV irradiation in the presence of TR was associated with the conversion of the hemeprotein to the high spin state. Cytochrome *c* high spin state is detectable by direct EPR of the heme iron and, for the first time, it was characterized by MCD. The concentration of cytochrome *c* samples was a limiting factor in detecting a heme iron EPR signal. Therefore, all EPR data were obtained at a high protein concentration (100 µmol/L). However, in the presence of a high protein concentration, 25 µmol/L TR was not able to cause damage in a population of cytochrome *c* enough to produce a detectable EPR signal of an oxidized protein fraction (not shown). Therefore, under the experimental conditions required for the detection of a confident heme iron EPR signal, 200 µmol/L TR was used. The UV exposure of 100 µmol/L cytochrome *c* in the presence of 200 µmol/L TR converted a fraction of the protein to the high spin state with a detectable EPR signal. Figure 4A shows the EPR spectrum (X-band) of 100 μmol/Lcytochrome *c* in a 5 m mol/Luniversal buffer, pH 4.0, at 11 K, a condition in which cytochrome *c* is in the native form. This spectrum is assigned to the well-known Fe(III) low-spin form with a rhombic symmetry that displays signals at g_//_ = 3.07 and g_⊥_ = 2.23. After 120 min of irradiation, the EPR signal of a low spin Fe(III) form was changed to a high spin Fe(III) form with axial symmetry with g = 6.0 [18] (Figure 4B). This result is consistent with the loss of the heme iron sixth coordination due to oxidation of the Met80 residue. At 2.5 mmol/LTR, the conversion of cytochrome *c* to the high spin form was not observed (not shown), and the signal of a TR cation radical could be detected (Figure 4C). The EPR spectrum of a stable TR cation radical photo-generated in the absence of cytochrome *c* (Figure 4C, gray line) was identical to the result obtained in the presence of the protein (Figure 4C, black line). This result is consistent with the UV protection of cytochrome *c* and reinforces the idea that cytochrome *c* does not interfere with the stability of a TR cation radical. The absence of significant signal g = 4.3 indicates that the porphyrin ring was preserved from the oxidation [19]. Considering that only the EPR signal of the high spin form was detected, it was important to know whether, in this condition, native cytochrome *c* was totally converted to the high spin state. Thus, samples of cytochrome *c* plus TR, in the same conditions used for EPR experiments, were irradiated and analyzed by magnetic circular dichroism (Figure 4D). The spectrum of 100 µmol/Lcytochrome *c* exposed to UV light in the presence of 200 mmol/LTR is a composite of the cytochrome *c* Fe^2+^ spectrum and of a modified form of the protein (gray line). The subtraction of the contribution of the signal of native EPR silent Fe^2+^ cytochrome *c* (dashed line) rendered the MCD spectrum of the modified high spin form (cytc405) that is shown as the black line. Before subtraction, the spectrum of 100 µmol/L Fe^2+^ native cytochrome (dashed line) was multiplied by the factor = 2.68 to render the MCD Q_α_ band with the same intensity of that exhibited by the fraction of cytochrome *c* that was photo-reduced by TR. At this point, it is important to consider the possible mechanism involved in the UV light- and phenothiazine-promoted cytochrome *c* damage.

### The mechanism of cytc405 formation induced by UV light and phenothiazines

The irradiation of cytochrome *c* in a buffered solution of D_2_O, at pD 4.4 (not shown), did not affect significantly the rate of a UV light- and phenothiazines-promoted Soret band blue shift. This result suggests that singlet oxygen [O_2_(^1^ Δ_g_)] was not the principal pro-oxidant species responsible for the oxidative damage in the absence and in the presence of phenothiazines. In the absence of drugs, the sensitization of the phorphyrin ring by the UV light irradiation probably promotes the oxidation of Met80 that, in turn, reacts with ground state molecular oxygen and generates the sulfoxide derivative. The irradiation of cytochrome *c* in the presence of different phenothiazine concentrations and at different pH values revealed that the predominant formation of ferrous cytochrome *c* (protection against UV light irradiation) or cyt*c*405 (oxidative damage) depends of a balance of some factors. These factors are: the pH-dependent efficiency of native cytochrome *c* reduction by phenothiazines and reoxidation pro-oxidant species; the pH-dependent photochemistry of the drugs; and the rate of Met80 oxidation to sulphoxide derivative. The latter event generates a high spin cytochrome *c* whose ferrous form does not exhibit distinguishable α and β Q bands [19]. Consistently, a discrete cytochrome *c* reduction could be detected in the early stages of irradiation in conditions where the oxidative damage was mild. For example, at pH 4.0, with 5 µM of drugs (not shown), and in conditions in which the reduction and stability of native cytochrome *c* and the deactivation of photo-generated cation radicals are favored, i.e. at pH 8.0 with 25 μmol/LTR, the reduction was observed (Figure 2F). As previously mentioned, the UV light irradiation of cytochrome *c* with low concentrations of phenothiazines at pH 8.0 led to protection of the protein against photo-damage and increased the turbidity of the samples. Literature data [29,37] and our laboratory results (unpublished) have demonstrated that phenothiazinescation radicals can oligomerize and form nanostructures in the presence and in the absence of polymers such polyethylene glycol. Consistently, in the conditions in which a reduced form of cytochrome *c* was detected by UV-visible spectroscopy, a significant increase of baseline was also present (Figures 2C, 2F, 3B and 3E, dotted lines). These conditions were all consistent with the oligomerization of phenothiazines. At high phenothiazine concentrations (Figures 2C and 3B), the aggregation of photo-generated cation radicals stabilizes this species, preventing radical pairs recombination and oxidative attack of cation radicals on cytochrome *c*. In addition, the TR^•^ and FP^•^ species with long lives are able to transfer electrons to heme iron. The ferrous form of cytochrome *c* is the principal target of the population of phenothiazine cation radicals that did not became stabilized in the aggregates or did not oligomerize. Simultaneously, the high concentration of phenothiazines filtered the UV light, and the protein is preserved from light damage and oxidative attack of the phenothiazine cation radical. At pH 8.0, despite low concentrations of phenothiazine (Figures 2F and 3E) cytochrome *c* protection against UV-promoted oxidative damage that converts cytochrome *c* to cyt*c*405, protection was also observed. Consistently, high turbidity was also present in this condition. This result suggests that phenothiazine oligomerization promoted by UV light irradiation also occurrs in alkaline pH. Therefore, faster oligomerization of cation radicals impairs the oxidative attack to cytochrome *c* as well as the efficient reoxidation of ferrous cytochrome *c* formed by the reducing TR^•^ and FP^•^ species. On the other hand, the UV protection observed at pH 8.0 in the presence of 25 µmol/L phenothiazine is not a warranty that phenothiazines are efficient UV filter in this concentration, since the reduction of heme iron by TR^•^ and FP^•^ species prevents the oxidative attack to met80. The result is cytochrome *c* protection against the oxidation of the heme iron sixth ligand and the net yield of ferrous cytochrome *c*.

**Figure 5:**
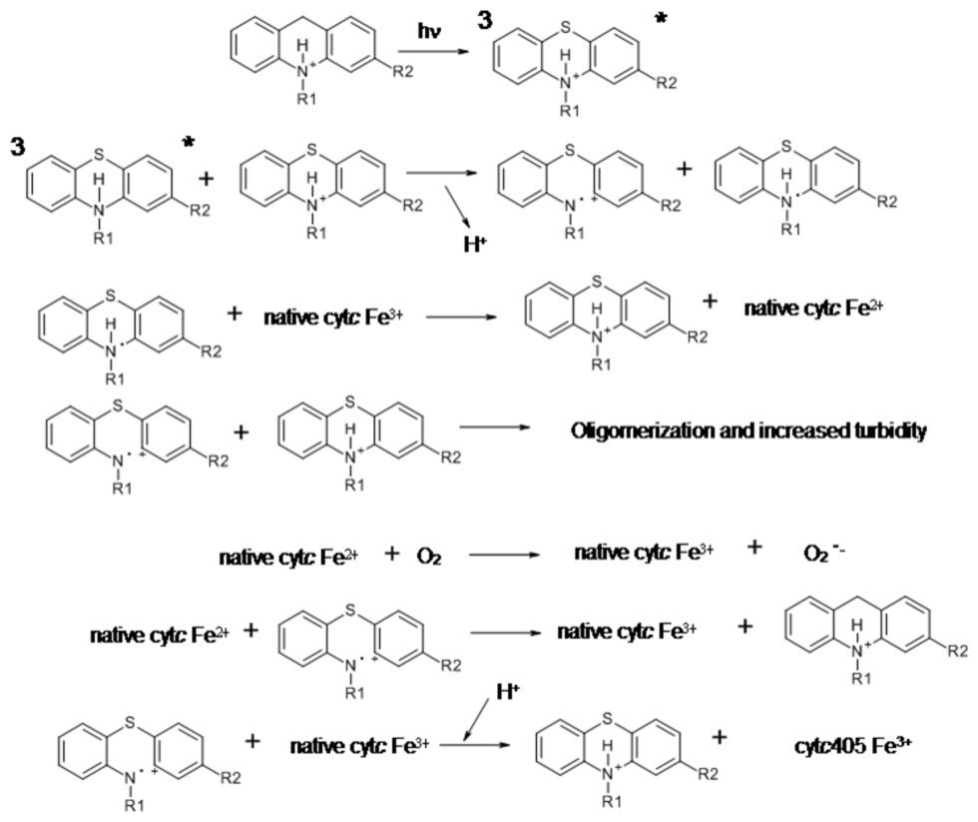
Proposed mechanism involved in the modifications of cytochrome c and phenothiazines submitted to UV-irradiation.

A similar mechanism has been observed when cytochrome *c* was exposed to photo-chemically excited MB^+^ at pH 8.0 [19]. Figure 5 shows the proposed mechanism to the formation of cyt*c*405 promoted by the irradiation of phenothiazine derivatives.

### Photochemically-generated TR cation radical attacks cytochrome *c* side chain amino acids

The MALDI-ToF mass spectrometry analysis of cytochrome *c* fragments obtained by digestion with trypsin, exposed to photo-chemically generated TR cation radicals, revealed, after 120 min of irradiation, a mass increase of 16 units in the fragment containing amino acid residues from 56 to 73 (Table 1). In this fragment, both Met65 and Tyr67 are candidate targets for the pro-oxidant species. However, it is more probable that the oxidation process occurred in Met65 since it is more accessible in the cytochrome *c* structure. In this case, the mass increase of 16 units was compatible with the formation of a sulphoxide derivative (Table 1). Besides the Met65 oxidation, the protein fragments containing oxidized Met80 and Tyr74 (an increase of 16 units) were also observed after the trypsin digestion of samples subjected to irradiation for 120 min (Table 1). As previously observed, the MALDI-ToF analysis and SDS-Page of non-digested cytc405 (not shown) discarded the possibility of protein fragmentation.

**Table 1:** Identifiable tryptic fragments of cytochrome *c* in the presence of TR before and after irradiation, as detected by MALDI.

**Mass (0 s)**	**Comp. Mass**	**Error (m/z)**	**Res. start**	**Res. end**	**Mass (30 s)**	**Mass (5 and 120 min)**	**Peptide sequence**
1305.725	1350.693	0.0326	89	99	1350.693	1305.759	GEREDLIAYLK
1632.637	1632.812	-0.1742	9	22	1632.637	1632.649	IFVQKCAQCHTVEK
1433.013	1432.769	0.0369	26	38		1432.806	HKTGPNLHGLFGR
1297.51	1297.51		28	39		1297.949	TGONLHGLFGRK
2083.135	2083.8		5	72		2083.135	GITWKEETLMEYLENPK
908.744	908.74		80	87		908,744*	MIFAGIKK
1434.902	11434.65		26	38		1434.902	HKTGNIHGLFGR
1634.74	1634.94		9	22		1634.704	IFVQKCAOCHTVEK
1295.807	1295.710	0.0968	28	39	1295.710	1295.755	TGPNLHGLFGRK
1018.566	1018.45		14	22		1018.566	CAQCHTVEK
**906.702**	**906.536**	**0.1660**	**80**	**87**	**906.702**	**922.692^c^**	**MIFAGIKK**
**1437.750**	**1437.805**	**-0.0553**	**74**	**86**		**1470.810**	**YIPGTKMIFAGIK**
**1623.79**	**1623.79**		**61**	**73**		**1657.753^a^**	**EELMEYLENPKK**
**1092.63**	**1092.63**		**92**	**100**		**1110.659**	**ADLIAYLKK**
**634.39**	**634.39**		**9**	**13**		**650.243**	**IFVQK**
**678.375**	**678.375**	**-0.1997**	**74**	**79**		**693.102^b^**	**YIPGTK**

* Mass of the peptide fragment indicates that Met80 was not oxidized.

*a* Increase in the expected mass of the peptide fragment compatible with the oxidation of Met65.

*b* Increase in the expected mass of the peptide fragments indicates the oxidation of Tyr74.

*c* Increase in the expected mass of the peptide fragment compatible with the oxidation of Met80.

Text in bold highlights the fragments in which changes were found.

Selected protein: Protein information: gi, 117963, sp, P00006, CYC_BOVIN – Cytochrome *c*, rank: 1. Expectation: 0.000 Coverage: 79.8, pl: 9.7 Mass: 11.55 kDa. Matched peptides: 13 Measured peptides: 16.

Data were obtained as described in Materials and Methods. Residue number is based on the sequence of the mature protein.

As previously described for cytc405 generated by excited MB^+^, the high spin form of cytochrome *c* generated by phenothiazines exhibited the capacity to be reduced by thiol groups, but not by diphenylacetaldehyde and excited thiazines.

## Conclusion

The irradiation of cytochrome *c* and phenothiazines in a homogeneous medium generated the phenothiazine-derived cation radicals (Type I mechanism). In the presence of low phenothiazine concentrations at an acidic pH, the monomer form of the drug and the photo-chemically-generated cation radical became prone to attack the protein. In high concentrations of phenothiazines, the stabilization of the cation radical present in the pre-micellar and micellar aggregates impaired the damage of the protein. In this condition, stabilization can also be achieved by the oligomerization of the drugs leading to the formation of nanoparticles that was corroborated by SEM images. The detection of a cytochrome *c* reduction (Figures 2 and 3) in specific conditions implies that TR^•^ and FP^•^ species similar to MB^•^, which was formed as shown in Figure 5, were able to transfer one electron to the cytochrome *c*heme iron [19]. In this case, the electron transfer from TR^•^ and FP^•^ species to the cytochrome *c*heme iron was likely dependent on the yield of these radicals and the protonation state of tyrosine residue. This was previously observed in the process mediated DPAA, which, as expected, was unable to reduce cyt*c*405 [27]. It is necessary to also consider the effect of pH on the quantum yield of^3^TR^+^* and ^3^FP^+^*.

As commented in the Introduction section, the photosensitivity associated to the therapeutic use of phenothiazines is well known, however, the present study demonstrates that photodamage can be changed to photoprotection by the aggregation of the phenothiazines molecules. The aggregation prevents the oxidative damage by stabilizing the cation radical and/or leading to a subsequent oligomerization and formation of nanostructures. In this condition, the UV absorption is maintained and photoprotection against direct UV damage prevails. In the present study, the concentration of cytochrome *c* and phenothiazines used in the absorbance spectroscopy experiments are within of the concentrations feasible to be found in cells. Sharonov et al, 2005 reported that the minimum concentration of horse heart cytochrome *c* to activate apoptosis was estimated to be 2.7 +/- 0.5 mmol/L (47 +/- 9 fg/cell) [20,38,39]. The aggregation of phenothiazines is dependent of two factors: drug concentration and the well-known interaction with biological membranes [40]. Regarding the concentrations of phenothiazines achieved in vivo, Amaral et al, 2001 and Ordway et al, 2002 reported intracellular values between 0.01 and 0.1 mg/L (0.24-2.4 µmol/L), however these drugs are concentrated up to 100 fold (24-240 µmol/L) in the lysosomes of macrophages [13,22,23,41-44]. Therefore, the aggregation of TR and FP is not favoured in the most of cells due to the concentrations and principally by the presence of lipid bilayers. These are probably the biological conditions contributing for the side effects of phenothiazines. According to the above-mentioned, the photoprotective effect of phenothiazines might be achieved only by an external use in conditions in which the drugs are maintained aggregated and impaired to be absorbed by the cells.

In the present study, by using cytochrome *c* as a biological model molecule, it was possible to demonstrate that photosensitivity and UV-protection by phenothiazines can be modulated by pH and the aggregation states of the drugs. In addition, the present study introduces the capacity of phenothiazines to oligomerize and form nanoparticle in a simple one step method and whose mechanism is under investigation in our laboratory.
